# Single-cell evidence for plasmid addiction mediated by toxin–antitoxin systems

**DOI:** 10.1093/nar/gkae018

**Published:** 2024-01-15

**Authors:** Nathan Fraikin, Laurence Van Melderen

**Affiliations:** Bacterial Genetics and Physiology, Department of Molecular Biology, Faculté des Sciences, Université Libre de Bruxelles (ULB), 6041 Gosselies, Belgium; Bacterial Genetics and Physiology, Department of Molecular Biology, Faculté des Sciences, Université Libre de Bruxelles (ULB), 6041 Gosselies, Belgium

## Abstract

Toxin–antitoxin (TA) systems are small selfish genetic modules that increase vertical stability of their replicons. They have long been thought to stabilize plasmids by killing cells that fail to inherit a plasmid copy through a phenomenon called post-segregational killing (PSK) or addiction. While this model has been widely accepted, no direct observation of PSK was reported in the literature. Here, we devised a system that enables visualization of plasmid loss and PSK at the single-cell level using meganuclease-driven plasmid curing. Using the *ccd* system, we show that cells deprived of a *ccd*-encoding plasmid show hallmarks of DNA damage, *i.e*. filamentation and induction of the SOS response. Activation of *ccd* triggered cell death in most plasmid-free segregants, although some intoxicated cells were able to resume growth, showing that PSK-induced damage can be repaired in a SOS-dependent manner. Damage induced by *ccd* activates resident lambdoid prophages, which potentiate the killing effect of *ccd*. The loss of a model plasmid containing TA systems encoding toxins presenting various molecular mechanisms induced different morphological changes, growth arrest and loss of viability. Our experimental setup enables further studies of TA-induced phenotypes and suggests that PSK is a general mechanism for plasmid stabilization by TA systems.

## Graphical Abstract

**Graphical Abstract:**
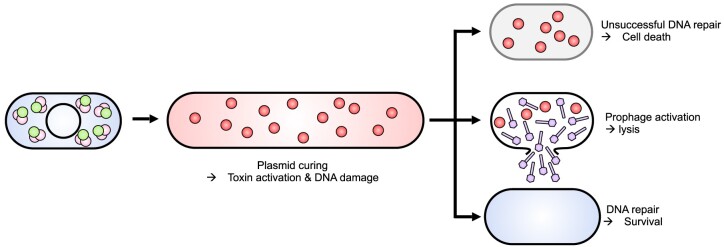

## Introduction

Plasmids are key drivers of genome evolution by promoting gene shuffling via horizontal gene transfer (1). In addition to cargo accessory genes that provide beneficial ecological traits under selective conditions, plasmids encode ‘core’ genes allowing for copy number control, multimer resolution and partitioning in daughter cells ensuring their stable maintenance in growing bacterial populations (2). Along with these maintenance functions, plasmids often encode toxin–antitoxin (TA) modules that are generally composed of two genes encoding a toxin and its cognate antitoxin (3–6). While these systems were originally discovered on the F and R1 plasmids in the 1980s (7–9), the combination of comparative genomics, biochemistry and structural biology led to the identification and characterization of dozens of such systems encoded in bacterial plasmids and chromosomes (3–6). TA systems turned out to be widespread, employing toxins with highly diverse activities and antitoxins of different nature and mode of action (4,10). The latter served as the basis for establishing TA system classification, and we currently distinguish eight different classes among which type I (RNA antitoxin inhibiting translation of its cognate toxin) and type II (protein antitoxin sequestering its cognate toxin) are the best characterized (4).

The first type II TA module was identified on the *Escherichia coli* F plasmid. Works from several groups showed that this large conjugative plasmid encodes a two-gene locus (*ccd* for control of cell division) that prevents plasmid loss (8,9). This type II TA system is composed of the CcdA antitoxin and CcdB toxin (9). An initial model proposed that upon decrease of F copy number, inhibition of cell division by CcdB would allow plasmid replication and restoration of the appropriate number of segregational units to ensure successful plasmid partitioning in nascent daughter cells, therefore coupling plasmid replication to cell division (9). Population-level studies using a thermosensitive replicon carrying the *ccd* system revealed a plateauing in viable cell counts at non-permissive temperature, indicating that this locus could inhibit growth in plasmid-destabilizing conditions (9,11). However, total cell counting under these conditions revealed a large population of non-viable cells concomitantly with filamentation, a phenotype reminiscent of DNA damage and induction of the SOS response (11,12). Separation of filamentous cells by Percoll gradient showed that these cells are not viable, suggesting that *ccd* triggers DNA damage and cell death in plasmid-free segregants (11). This locus was ultimately named *ccd* for control of cell death (13). Subsequent work established that the CcdB toxin poisons DNA gyrase, leading to the formation of double-stranded DNA breaks (DSBs) and induction of the SOS response, supporting the filamentation phenotype (13,14). Simultaneously to the discovery of the *ccd* system, another TA system was identified on the R1 plasmid (7). The type I *hok-sok* system was shown to comprise an antitoxin RNA (*sok*, suppression of host killing) that inhibits translation of the toxin (*hok*, host killing) (7). Using comparable replication-thermosensitive replicons, the *hok-sok* locus was found to induce a plateau in viable cell counts at non-permissive temperature, in an analogous manner to the *ccd* locus (7). This was accompanied with a loss of cytosolic content, which indicated that this system triggers ghost cell formation in plasmid-free segregants through a molecular mechanism that differs from *ccd* (7). It was subsequently showed that the Hok toxin is a small pore-forming protein that inserts into the inner membrane and induces loss of cytosolic content (15). These pioneering studies laid the formulation of the post-segregational killing (PSK) model or addiction as quoted later on, in which TA systems favor plasmid retention in populations by inducing cell death in plasmid-free segregants (7,11,16). It was further shown that the molecular basis of addiction relies on different stability between the components, antitoxins being labile (17–19). While antitoxins and toxins are constantly replenished in plasmid-containing cells, loss of TA-encoding genes in plasmid-free daughter cells would lead to the depletion of the unstable antitoxin, liberation of the toxin, and thus target corruption and cell death (17–19).

While the addiction model was elaborated from fragmentary observations, it became an accepted paradigm regarding the mechanisms by which TA systems promote plasmid retention (3–6). However, models in which TA systems regulate plasmid replication or segregation continued to be proposed throughout the years. For example, overexpression of the Kid toxin from plasmid R1 was shown to uncouple DNA replication and cell division to facilitate plasmid inheritance, as initially proposed for the *ccd* system (20). Another study showed that the omega–epsilon–zeta tripartite system from *Streptococcus* plasmid pSM19035 was able to regulate plasmid copy number through the Omega gene product, which encodes the repressor component of this tripartite TA system (21). Similarly, the PrpA antitoxin of the PrpAT system was shown to inhibit replication of the *Pseudoalteromonas* plasmid pMBL6842 by competing with the plasmid-encoded replication initiator for iteron sequences (22). However, in all those cases, conditions that alter regulator or antitoxin amounts are unclear, with the mechanism allowing these TA systems to control plasmid stability by ways other than PSK remaining elusive. Here, we used time-lapse fluorescence microscopy to re-examine the PSK model by following the plasmid loss events at the single-cell level in real-time. We first observed segregation of a fluorescent protein-tagged mini-F plasmid carrying the *ccd* locus. We provide direct evidence that this system does not prevent plasmid loss *per se*, but rather induces SOS response after plasmid loss, confirming that *ccd* is activated in plasmid-free segregants. To increase the loss frequency and facilitate our analysis, we engineered a unique system in which plasmid curing is forced through digestion by the I-SceI endonuclease. Using this system, we show that *ccd* triggers cell death in the majority of plasmid-free segregants, with 46% of cells being able to escape killing due to SOS-dependent repair of CcdB-induced DNA damage. Curing plasmids encoding six other TA systems presenting various toxicity mechanisms also resulted in cell death, although these systems were more lethal than *ccd* under our conditions. Altogether, our work establishes an experimental system that enables to study TA activation at the single-cell level. Our results showcase the first live observation of PSK and of cells that escape this killing mechanism. Activation of plasmid-encoded TA systems with various activities and targets systematically resulted in cell death, establishing TAs as cell death modules and PSK as a general mechanism that explains retention of TA-encoding replicons.

## Materials and methods

### Strain and plasmid constructions

Plasmids used in this study are detailed in Supplementary Table S1. Plasmids were constructed by standard restriction-ligation methods using T4 DNA ligase (NEB) or using the NEBuilder assembly kit (NEB). Polymerase chain reactions (PCRs) were performed using Q5 DNA polymerase (NEB) or PrimeSTAR MAX (Takara). Oligonucleotides primers used in this study are detailed in Supplementary Table S2.

The trackable mini-F vector that yielded pNF03 was constructed by ligating a synthetic mNeongreen-encoding gene (23) at the SacI and PacI sites of pJYB240 (24). pNF03*ccd* was constructed by restoring a frameshift in *ccdB* using primers ApaLI-ccdB F and ApaLI-ccdB R and then by digesting the PCR product with ApaLI and circularizing it. pNF03 was constructed by deleting the *ccdAB* operon using primers delccd F and delccd R.

The pNF04 plasmid, a trackable mini-F vector that can be cleaved by I-SceI, was constructed by amplifying a mini-F replicon with an mNeongreen-tagged SopB from pNF03 using primers NotI-pNF04bb F and HindIII-pNF04bb R, while a kanamycin resistance cassette was amplified from pUA66 (25) using primers HindIII-KmR F and NotI-KmR R. These two fragments were digested by NotI and ApaLI and ligated. An I-SceI cutting site was inserted into this vector using primers 04Sce F and 04Sce R, yielding pNF04.

The pSce plasmid, which allowed arabinose-induced production of I-SceI, was constructed using the NEBuilder assembly by assembling a fragment amplified from pDL2655 (26,27) using primers CmR-Sce F and CmR-Sce R and a pSC101 origin of replication amplified from pUA66 (25) using primers ori-Sce F and ori-Sce R, yielding pSce.

TA systems were cloned in the AatII and HindIII sites of pNF04 and pNF05 using the following primers on their respective templates: AatII-ccd F and HindIII-ccd R for *ccdAB* from pNF03*ccd*, AatII-vap F and HindIII-vap R for *vapBC* from *Shigella flexneri* M90T genomic DNA, AatII-par F and HindIII-par R for *parDE* from RH8000, AatII-doc F and HindIII-doc R for *phd-doc* from phage P1vir, AatII-hok F and HindIII-hok R for *hok-sok* from BW27873 R1^+^, AatII-hig F and HindIII-hig R for *higBA* from a synthetic gene derived from pRts1, and AatII-tac F and HindIII-tac R for *tacAT* from a synthetic gene derived from *E. coli* 53638.

All strains used were isogenic to the MG1655 clone used as wild-type strain, in which relevant alleles were transduced. FRT-flanked resistance cassettes were excised using *flp* expression from pCP20 when applicable (see Supplementary Table S3) (28).

### Plasmid stability assay in bulk cultures

Cells were grown to exponential phase in MOPS-maltose medium with 15 mg/ml chloramphenicol to maintain mini-F plasmids labeled with SopB-mNeongreen. At time 0, cells were diluted 1000× in MOPS-maltose medium without chloramphenicol and left to grow for cycles of 10 generations (12 h). After each 10-generation growth cycle, cultures were diluted 1000× in MOPS-maltose medium to maintain cells in exponential growth. After 10, 30 and 50 generations, snapshots of the cultures were taken and at least 1000 cells were counted. Plasmid loss rate was calculated as a linear regression of cells devoid of SopB-mNeongreen foci over generations.

### Time-lapse microscopy analysis

**Figure 1.**
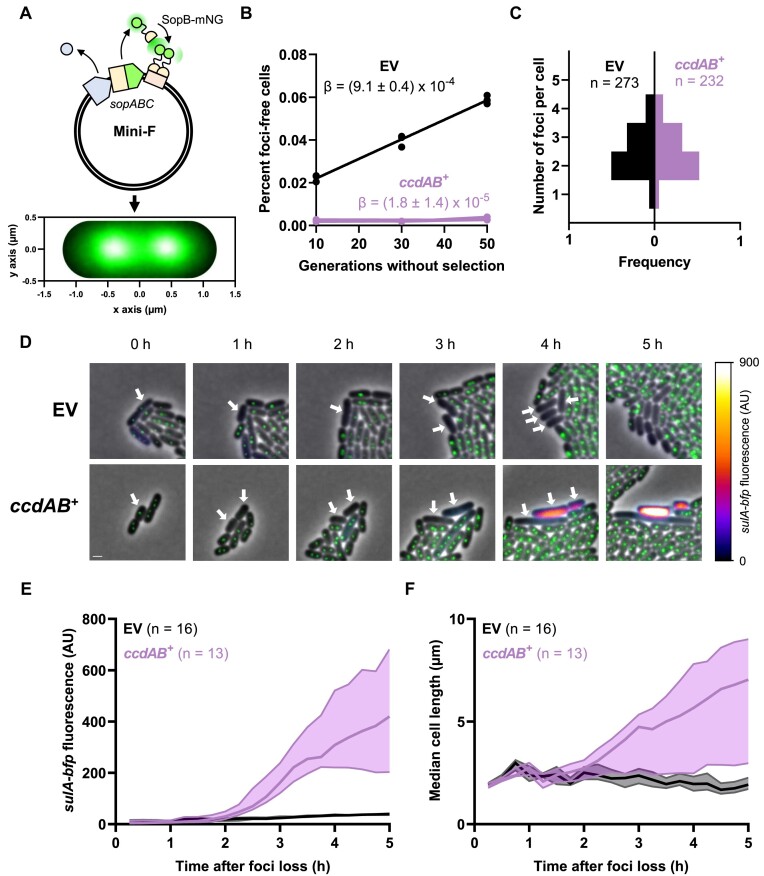
The *ccd* system induces the SOS response in plasmid-free segregants. (**A**) Illustration of the mini-F tracking system. Top: The SopB protein from the native partition system of F was fused to the mNeongreen fluorescent protein in the pNF03 mini-F plasmid. Binding of SopB-mNeongreen to the *sopC* centromere leads to the formation of green fluorescent foci that enables positional tracking of the plasmid. Bottom: Average SopB-mNeongreen fluorescence intensity signal of 115 cells displaying two SopB-mNeongreen foci. (**B**) Loss of mini-F plasmids in continuous culture. FN042 cells transformed with pNF03 (EV) and pNF03*ccd* (*ccdAB^+^*) were grown to exponential phase in MOPS medium containing 0.4% maltose and 15 μg/ml chloramphenicol to promote mini-F retention before being diluted 1000× in the same medium without antibiotic. Cells were diluted 1000× every 12 h and allowed to grow for 10 generations every cycle. The proportions of foci-free cells were counted at indicated time points and a linear regression was fit to the data of three independent replicates to obtain plasmid loss rate per generation (*β*). (**C**) Copy number of mini-F vectors. FN042 cells transformed with pNF03 (EV) and pNF03*ccd* (*ccdAB^+^*) grown on MOPS medium containing 0.4% maltose were imaged on agarose pads. The numbers of SopB-mNeongreen foci were counted for the indicated number of cells. (**D–F**) Time-lapse fluorescence microscopy analysis of unperturbed mini-F loss. FN042 cells transformed mini-F vectors pNF03 (EV) or pNF03*ccd* (*ccdAB^+^*) imaged by time-lapse fluorescence microscopy every 15 min on agarose pads made with MOPS medium containing 0.4% maltose. (**D**) Representative micrographs of mini-F loss events. The SopB-mNeongreen fusion that localizes the plasmid is shown in green, while the *sulA-bfp* fusion that reports the SOS response is shown by the associated color scale. Time 0 corresponds to the last time point where SopB-mNeongreen foci were visible in arrow-indicated cells. Scale bar is 1 μm. Quantification of median *sulA-bfp* fluorescence (**E**) and cell size (**F**) over time in the indicated number of plasmid-free segregants after foci loss. Shaded areas represent interquartile ranges.

Cultures for microscopy were prepared by diluting overnight cultures to OD_600nm_ 0.05 in MOPS medium (29) with indicated supplements. After reaching an OD_600nm_ of 0.5, the cultures were diluted 100× in the appropriate medium and either spotted on a sealed agarose pad (MOPS medium, 2% agarose, Figures 1D–F and 4A) or loaded in a CellASIC Onix plate (Merck) and perfused with MOPS medium containing 0.4% arabinose and 20 μg/ml chloramphenicol at 34.5 psi (Figures 3A and 5C). Microscopy experiments were performed using an Axio Observer Z1 microscope (Zeiss) equipped with a heating chamber, a motorized stage, an LED illumination system (Colibri 7, Zeiss) and an sCMOS camera (ORCA-Flash4.0 V2, Hamamatsu). mTagBFP2 was imaged using a 430 nm LED (12% intensity, 500 ms exposure), a 405/40 nm excitation filter and a 455/50 nm emission filter (Chroma). mNeongreen was imaged using a low-power 511 nm LED (50% intensity, 2000 ms exposure), a 480/40 nm excitation filter and a 535/50 emission filter (Filterset 49011,Chroma). mCherry was imaged using a 590 nm LED (50% intensity, 500 ms exposure), a 530–585 excitation filter and a 615 nm longpass emission filter (Filterset 00, Zeiss). Images were taken every 15 min. Cells were outlined and segmented using MicrobeJ (30), with median intensity of fluorescence channels used as measurements of fluorescence after subtraction of the camera offset (100 bits). All quantifications are displayed with time 0 of each individual loss event being the last time point where a SopB-mNeongreen focus is visible.

### Plasmid retention assay by flow cytometry

Overnight cultures grown in MOPS medium with kanamycin 25 μg/ml were diluted to OD_600nm_ 0.05 in the same medium and grown to exponential phase (OD_600nm_ 0.5). These cultures were then diluted 1000× in MOPS medium without kanamycin and grown for indicated times before being processed by an Attune Nxt Flow cytometer. Green fluorescence was measured using a 488 nm laser and a 522/31 emission filter (Omega Filters). Cells were gated empirically to remove background signal and cell doublets were filtered out based on their higher side-scattering pulse area-to-height ratio.

### PSK assay on plates

Overnight cultures grown in MOPS medium supplemented with 0.4% glucose, 20 μg/ml chloramphenicol and 25 μg/ml kanamycin at 37°C were diluted to OD_600nm_ 0.05 in the same medium and grown at 37°C. At OD_600nm_ of 0.5, cells were serially diluted in phosphate-buffered saline and plated on M9 plates (22 mM KH_2_PO_4_, 48 mM Na_2_HPO_4_, 18.8 mM NH_4_Cl, 8.6 mM NaCl, 2 mM MgSO_4_) supplemented with either 0.1% glucose and 0.3% arabinose to induce plasmid curing or 0.4% glucose and 25 μg/ml kanamycin to promote plasmid retention. These plates were then incubated for 30 h at 37°C before colony counting.

### Real-time quantitative PCR

Samples of 1 ml were taken from cultures grown to OD_600nm_ of 0.5 and centrifuged (5000 RCF, 5 min). After washing with 0.9% NaCl, cell pellets were resuspended in 100 μl lysis buffer (10 mM Tris–HCl, pH 8.0, 1 mM EDTA, 1% Triton X-100, 0.5% Tween 20) and boiled on a heating block for 10 min. Debris was pelleted (10 000 RCF, 2 min) and supernatants were conserved at −20°C until further use. PCR mixes were prepared using iTaq SYBR Green Master Mix (Bio-Rad) and dispatched in reaction volumes of 25 μl containing 10% (2.5 μl) of supernatant. Primers to amplify the F-encoded *repE* gene (repE F and repE R) or the chromosome-encoded *dnaN* gene (dnaN F and dnaN R) were used at 300 nM (Supplementary Table S2). Amplification was quantified in real time by detecting DNA-bound SYBR Green on the FAM channel of a CFX1000 instrument (Bio-Rad) and cycle thresholds (Cq) were detected using the Maestro software (Bio-Rad).

## Results

### Steady-state loss of mini-F plasmids reveals activation of the *ccd* TA system in plasmid-free segregants

We first examined the unperturbed segregation of a fluorescently labeled mini-F replicon at the single-cell level. This replicon is maintained at two copies per chromosome equivalent (31), and is actively segregated by the *sopABC* system, a type Ia partition system. SopB binds the *sopC* centromeric repeats to form partition complexes, which are segregated through the ATPase activity of SopA (32,33). We fused SopB to mNeongreen, a bright monomeric fluorescent protein, allowing to follow partition complex localization with minimal perturbation of plasmid segregation as described previously (Figure 1A) (34,35). Fluorescence was localized in foci along the medial axis of cells, confirming the functionality of our reporter (Figure 1A) (34,35). In bulk cultures, this plasmid was lost at a rate of 0.09% per generation, which is comparable to what was observed with other mini-F derivatives where SopB is tagged with a fluorescent protein (Figure 1B) (34,35). Introduction of the *ccd* system in this plasmid leads to undetectable loss levels, confirming the plasmid-stabilizing property of this system (Figure 1B). To rule out any effect of *ccd* on segregational stability through an increase in plasmid copy number, the number of SopB-mNeongreen foci per cell as a proxy for plasmid copy number was quantified. In good adequation with the established literature (31), we found an average foci number of 2.48 per cell, with no difference detected between an empty vector and a *ccd*-encoding plasmid (Figure 1C). DNA quantification by real-time PCR confirmed that *ccd* does not affect the copy number of our mini-F model plasmid (Supplementary Figure S1).

Using this SopB fusion as a proxy for plasmid residency, we grew microcolonies of plasmid-containing cells on agarose pads over the course of 3.2 × 10^4^ live divisions for cells carrying a *ccd*-deficient mini-F plasmid and for 2.9 × 10^4^ divisions of cells carrying a *ccd*-encoding plasmid. Similar numbers of division events resulting in plasmid-free segregants were observed for the empty and *ccd*-encoding plasmids (29 and 31, respectively), suggesting that *ccd* does not affect the segregation of its replicon. A total of 16 *ccd*-deficient and 13 *ccd*-positive plasmid-free segregants were imaged in optimal conditions. We quantified cell length and induction of the SOS response, using a *sulA-bfp* transcriptional reporter for the latter (Figure 1D) (36). A gradual increase in BFP fluorescence was observed after 2 h in cells that lost a *ccd*-encoding plasmid but not in those carrying an empty vector, confirming that the SOS response is induced post-segregationally (Figure 1E). Filamentation, a hallmark of the SOS response (37), was observed in *ccd* plasmid-free segregants, synchronously with *sulA-bfp* induction (Figure 1D). Cells reached a median cell length of 7 μm 5 h after plasmid loss, compared to 2 μm for cells that lost an empty plasmid (Figure 1F). Our results therefore demonstrate that *ccd* does not prevent plasmid missegregation but rather triggers DNA damage that leads to induction of the SOS response in plasmid-free segregants. Moreover, our single-cell data showcase the first live evidence of TA system activation in conditions where toxin concentrations are not ectopically manipulated.

### Meganuclease-mediated curing of mini-F plasmids induces *ccd*-mediated killing

**Figure 2.**
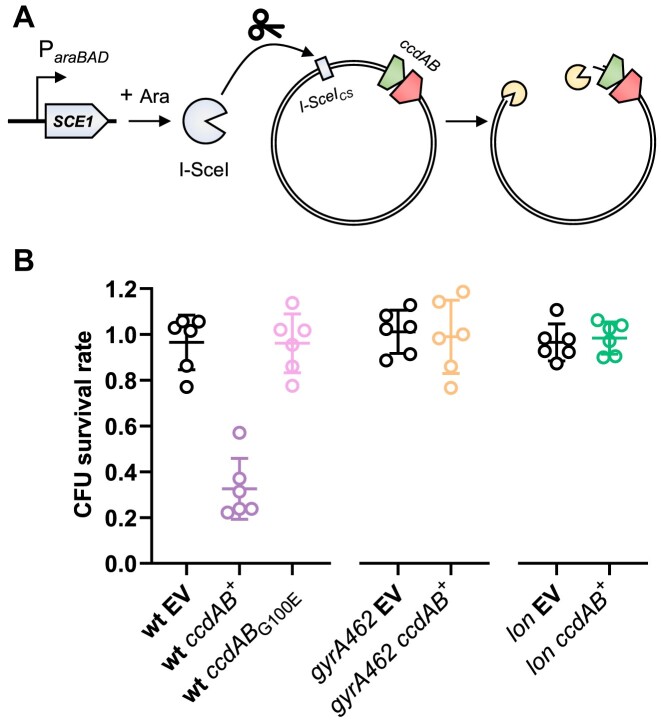
I-SceI-mediated curing reveals PSK. (**A**) Schematic representation of the plasmid curing system. The *SCE1* gene is under the control of the *araBAD* arabinose-inducible promoter on a low-copy plasmid (pSce), which allows production of the I-SceI restriction enzyme when cells are grown in the presence of arabinose. Plasmid pNF04, which is used to clone TA systems, contains an I-SceI cutting site (*I-SceI_CS_*), which allows its digestion and curing by the restriction enzyme when arabinose is added to the medium. (**B**) Viability loss by *ccd* under plasmid curing conditions. Cells transformed with pSce and pNF04 derivatives (pNF04, EV; pNF04ccd, *ccdAB^+^*; pNF04*ccdGE*, *ccdAB_G100E_*) were grown to exponential phase in MOPS medium containing 0.4% glucose, 25 μg/ml kanamycin and 20 μg/ml chloramphenicol, serially diluted and spotted on M9 plates containing 20 μg/ml chloramphenicol and either 0.4% glucose and 25 μg/ml kanamycin to promote plasmid retention or 0.1% glucose and 0.3% arabinose to promote plasmid curing. Data represent the mean and standard deviation of three independent experiments.

Because steady-state loss of the mini-F plasmid is a rare occurrence, plasmid-free segregants quickly get outcompeted and drowned in plasmid-bearing siblings, rendering tracking of their fate difficult. Indeed, while the aforementioned setup enabled us to visualize *ccd* activation and induction of the SOS response after plasmid loss, it failed to provide information regarding the viability of these plasmid-free segregants. To circumvent these limitations, we devised a system to induce synchronous plasmid curing in the whole population. We introduced an I-SceI cutting site on a mini-F plasmid and provided an arabinose-inducible *SCE1* gene *in trans* (Figure 2A). In this setup, addition of arabinose induces production of I-SceI, which specifically recognizes its cognate cutting site on the plasmid, leading to plasmid cleavage and degradation by endogenous exonucleases such as RecBCD (26). The absence of Chi sites in this plasmid should also prevent loading of RecA by RecBCD and therefore limit plasmid recombinational repair and induction of the SOS response (26). Quantitative PCR showed a 99.8% decrease of plasmid DNA levels 3 h after arabinose induction of SCE1, confirming the functionality of our plasmid curing system (Supplementary Figure S2). Curing an empty plasmid did not affect viability since plating cells on medium containing arabinose did not decrease colony-forming units compared to medium containing glucose and kanamycin, showing that our plasmid curing system has a minimal impact on cell growth and viability (Figure 2B). On the other hand, curing a *ccd*-encoding plasmid led to a 68% decrease in cell viability, showing that while loss of a *ccd*-encoding plasmid induces cell death in most of the population, a significant proportion of the cells escape the toxic activity of CcdB (Figure 2B). Abolishing the toxicity of CcdB by substituting its 100th residue from glycine to glutamate (14,38) or by using a *gyrA462* mutant, which is not intoxicated by CcdB (13,14), restored viability and confirmed that DNA gyrase poisoning is required for *ccd*-induced PSK (Figure 2B). Likewise, *ccd*-induced killing after curing is abolished in a *lon* mutant, confirming the requirement of CcdA degradation by Lon to enable PSK as previously suggested (Figure 2B) (17).

Altogether, by specifically triggering plasmid curing we provide direct evidence that *ccd* is activated in plasmid-deprived cells, eliminating most of this population and thereby increasing plasmid retention at the population level. Moreover, we validate that plasmid curing and TA activation can be induced *à la carte* by I-Sce-mediated cleavage, therefore rendering this experimental setup highly suitable and attractive to study TA activation at physiological concentrations and identify factors regulating this process.

### Live imaging of plasmid curing reveals *ccd*-mediated PSK

**Figure 3.**
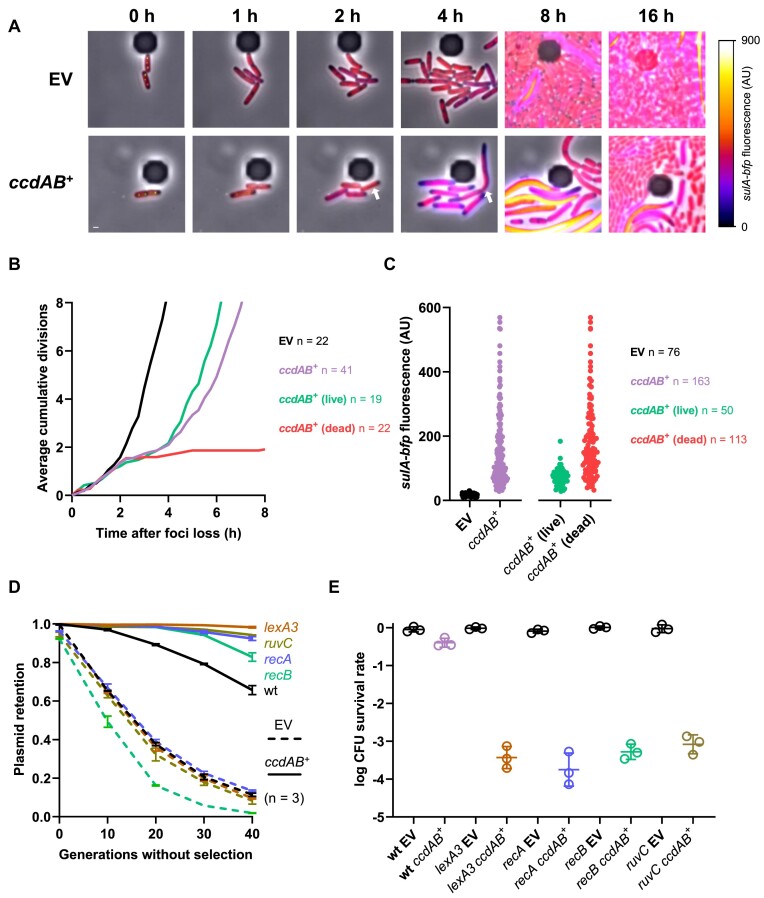
Live imaging of *ccd*-mediated PSK. (A–C) Time-lapse fluorescence microscopy analysis of I-SceI-mediated plasmid curing. FN053 cells were transformed with pSce and either pNF04 (EV, top) or pNF04*ccd* (bottom) plasmids, grown on MOPS medium containing 0.4% glucose, 25 μg/ml kanamycin and 20 μg/ml chloramphenicol, then loaded on microfluidic chips, perfused with MOPS medium containing 0.4% arabinose and 20 μg/ml chloramphenicol to induce plasmid curing and imaged by time-lapse fluorescence microscopy every 15 min. (**A**) Representative micrographs of plasmid-free segregants following I-SceI-mediated curing. SopB-mNeongreen is shown in green, the HU-mCherry fusion that localizes the chromosome is shown in red and the *sulA-bfp* reporter is shown with the associated color scale. A cell surviving the effects of *ccd* is shown by a white arrow. Scale bar is 1 μm. (**B**) Quantification of divisions by microscopy after plasmid loss. The number of cumulative divisions for observed plasmid loss events was quantified at each 15 min time point after SopB foci loss, with time 0 as the last time point where foci were detected, and averaged over the indicated number of imaged plasmid loss events (*n*). Fate of daughter cells from plasmid curing events was classified as dead (failure to form microcolonies) or live (formation of microcolonies). (**C**) Quantification of *sulA-bfp* median fluorescence intensity in daughter cells of plasmid-free segregants 4 h after SopB foci loss. (**D**) Plasmid retention in DSB repair-deficient mutants. Indicated strains transformed with pNF06 (dashed lines) or pNF06*ccd* (solid lines) were continuously grown in MOPS medium containing 0.4% glucose and sampled at indicated times during exponential phase to measure the proportion of fluorescent cells by flow cytometry. Data represent the mean and standard deviation of three independent experiments. (**E**) Viability of DSB repair-deficient mutants under plasmid curing conditions. Indicated mutants transformed with pSce and either pNF04 or pNF04*ccd* were grown to exponential phase in MOPS medium containing 0.4% glucose, 25 μg/ml kanamycin and 20 μg/ml chloramphenicol, serially diluted and spotted on M9 plates containing 20 μg/ml chloramphenicol and either 0.4% glucose and 25 μg/ml kanamycin to promote plasmid retention or 0.1% glucose and 0.3% arabinose to promote plasmid curing. Data represent the geometric mean and standard deviation of three independent experiments.

To further investigate the phenotypic changes brought by *ccd* upon plasmid curing, the plasmid curing system used above was adapted for use in time-lapse microscopy analysis. By growing cells with arabinose to induce I-SceI production, a progressive loss of SopB-mNeongreen foci produced by our model mini-F plasmid can be observed (Figure 3A and Supplementary Figure S3). We imaged the growth of 122 microcolonies during SCE1 induction, of which 67% had lost the SopB foci after 1 h, with >50% of loss events taking place between 30 and 60 min after arabinose addition. After 3 h, 100% of these microcolonies had lost their foci, confirming that I-SceI production induces plasmid loss at the single-cell level and that our setup can robustly induce plasmid curing in an entire bacterial population (Supplementary Figure S3 and Supplementary Movie S1).

As a proxy for CcdB activity, we quantified cumulative divisions and induction of the *sulA-bfp* reporter in plasmid-free segregants after plasmid curing. Curing an empty plasmid did not affect cell growth, which continued to divide exponentially after arabinose addition (Figure 3A and B, and Supplementary Movie S2). After losing a *ccd*-encoding plasmid, cells kept on dividing normally for 2 h (Figure 3A and B). After this time point, division rate plateaued and *sulA* induction was detected in *ccd* plasmid-free segregants but not in cells that lost the control plasmid (Figure 3A–C). However, division rate accelerated after the third hour of induction, which reflected a resumption of division and growth in part of the population (Figure 3B). Out of 41 analyzed loss events of a *ccd*-encoding plasmid, 19 (46%) resulted in division resumption and formation of a microcolony after plasmid loss, underlying the survival of these plasmid-free segregants (Figure 3B). On the other hand, 22 (54%) of these plasmid-free segregants failed to resume division and to produce microcolonies (Figure 3B). Both populations had detectable blue fluorescence produced by the *sulA-bfp* reporter 4 h after plasmid loss, suggesting that CcdB induces DNA damage in both subpopulations (Figure 3C). The absence of *sulA-bfp* induction at this time point in cells that lost an empty plasmid (17 ± 5 AU) also confirmed that cleavage of our model plasmid did not induce the SOS response (Figure 3C). While *sulA-bfp* induction levels showed a broad distribution in the bulk of the population (151 ± 115 AU), surviving cells were consistently located at the lower end of this spectrum (73 ± 28 AU). A lower induction of the SOS response could indicate less DNA damage endured by surviving cells and repair of CcdB-induced DSBs (Figure 3C).

We then assessed whether the DSB repair machinery was involved in survival to *ccd*-mediated PSK. DSB repair first requires end resection by the RecBCD nuclease, loading of RecA on single-stranded DNA (ssDNA) and activation of the SOS response by RecA-mediated autocleavage of the LexA repressor (26,37,39). RecA-ssDNA nucleoprotein filaments mediate pairing of the break with a sister locus. Subsequent strand invasion and homology-mediated repair of the break generate Holliday junctions that need to be resolved by the RuvABC complex (40). We first measured the stability of a partition-deficient plasmid in mutants deficient for every step of this repair process, which include a non-inducible *lexA3* mutant as well as *recA*, *recB* and *ruvC* mutants. After 40 generations of culture without selection for plasmid retention, only 11% of the cells retained fluorescence (Figure 3D). A *ccd*-encoding plasmid showed better yet limited retention, with 66% of the cells remaining fluorescent after 40 generations of culture (Figure 3D). The empty vector was lost at similar rates in *lexA3*, *recA* and *ruvC* mutants, while loss was slightly accentuated in a *recB* mutant (Figure 3D). However, a *ccd*-encoding plasmid displayed better retention in all these mutants when compared to a wild-type strain, with all mutants, except *recB*, showing a plasmid retention >90% (Figure 3D), suggesting that survival from *ccd*-mediated PSK requires repair of DSBs. Consistent with these results, I-SceI-mediated curing of a *ccd*-encoding plasmid reduced viability by three to four orders of magnitude in *recA*, *recB*, *ruvC* and *lexA3* compared to a wild-type strain, while curing of an empty plasmid did not affect viability in these mutants (Figure 3E). We therefore show that *ccd* is activated in all plasmid-free segregants, in which it induces DNA damage. However, this activation only results in a partial elimination of cured cells. Mutants inactivated for the repair of DSBs are more efficiently killed by *ccd* by several orders of magnitude, suggesting that survival to *ccd*-mediated PSK is facilitated by the repair of CcdB-induced DNA lesions. Moreover, these data validate the functionality of our experimental system to study TA activation and subsequent phenotypes at the single-cell level.

### Cooperativity between *ccd* and lambdoid prophages

**Figure 4.**
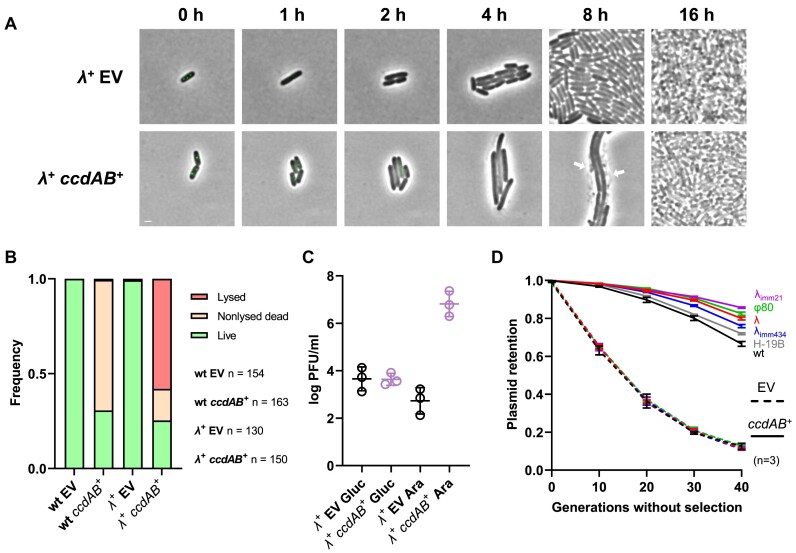
Cooperativity between *ccd* and lambdoid prophages. (A, B) Time-lapse fluorescence microscopy analysis of I-SceI-mediated plasmid curing in lambda lysogens. MG1655 cells were lysogenized with lambda and transformed with pSce and either pNF04 (EV, top) or pNF04*ccd* (bottom) plasmids, grown on MOPS medium containing 0.4% glucose, 25 μg/ml kanamycin and 20 μg/ml chloramphenicol, spotted on agarose pads with MOPS medium containing 0.4% arabinose and 20 μg/ml chloramphenicol to induce plasmid curing and imaged by time-lapse fluorescence microscopy every 15 min. (**A**) Representative micrographs of plasmid-free segregants following I-SceI-mediated curing. SopB-mNeongreen is shown in green, with time 0 as the last time point where foci were detected. Scale bar is 1 μm. (**B**) Quantification of survival and lysis in plasmid-free segregants in non-lysogen cells grown in microfluidics (as in Figure 3A) or lambda lysogens grown on agarose pads as described above. Fate of daughter cells from plasmid curing events was classified as Lysed (disappearance or loss of phase contrast), Nonlysed Dead (failure to form microcolonies) or Live (formation of microcolonies) in the indicated number of cells (*n*). (**C**) Production of viral particles by *ccd*-induced PSK. Lysogens transformed with pSce and either pNF04 (EV) or pNF04*ccd* were grown to exponential phase in MOPS medium containing 0.4% glucose, 25 μg/ml kanamycin and 20 μg/ml chloramphenicol, and diluted 100× MOPS medium containing 20 μg/ml chloramphenicol and either glucose (Gluc) or arabinose (Ara) as sole carbon source. PFUs were estimated from lysates after 16 h of growth. (**D**) Plasmid retention in lysogens. Lysogens of indicated phages transformed with pNF06 (dashed lines) or pNF06*ccd* (solid lines) were continuously grown in MOPS medium containing 0.4% glucose and sampled during exponential phase at indicated times to measure the proportion of fluorescent cells by flow cytometry. Data represent the mean and standard deviation of three independent experiments.

To further study *ccd*-induced phenotypes and illustrate the suitability of our experimental setup, we characterized the activation of *ccd* and its downstream consequences in backgrounds that carry lysogen lambdoid prophages, which are ubiquitous in *E. coli* but cured from our MG1655 lab strain. Since DNA double-stranded breaks are known to derepress lambdoid phages and induce the lytic production of viral particles (39), we investigated whether *ccd*-induced PSK in lambda lysogens could induce lysis and whether this lysis could, in turn, promote retention of a *ccd*-encoding plasmid in lysogens. While *ccd*-mediated PSK in a non-lysogen background triggered lysis in 0.6% of the population (Supplementary Movie S3), imaging of plasmid curing in lambda lysogens reveals a post-segregational induction of lysis in most cells that lost a *ccd*-encoding plasmid (58.0%) (Figure 4A and B, and Supplementary Movies S3 and S4). On the other hand, curing an empty plasmid in a lysogen background induced lysis in a neglectable fraction of plasmid-free segregants (0.8%), confirming that our plasmid curing system does not activate RecA (Figure 4B). Production of viral particles was quantified to confirm induction of the lytic cycle in *ccd* plasmid-free segregants. Lambda lysogens grown in MOPS glucose medium produced 10^3^–10^4^ plaque-forming units (PFUs) whether they were transformed with an empty or *ccd*-encoding plasmid (Figure 4C). Growth on arabinose to induce plasmid curing increased PFU yields by three orders of magnitude for cells that cured a *ccd*-encoding plasmid, but not for those that cured an empty plasmid, showing that loss of a *ccd*-encoding plasmid triggers entry into the lytic cycle followed by phage particle production (Figure 4C).

We next investigated whether lysogeny could enhance *ccd*-mediated PSK by inducing lysis in cells that would have otherwise survived intoxication by CcdB following plasmid loss. Our microscopy assays could detect a marginal reduction of survival to *ccd*-mediated PSK in lambda lysogens (25.3%) compared to non-lysogens (30.7%) (Figure 4B). However, plating on arabinose versus glucose medium failed to detect such a small difference, likely due to the intrinsic error of 10-fold dilutions used in this assay, which is more suited to detect differences on a logarithmic scale (Supplementary Figure S4). To robustly evaluate the effect of lysogeny on PSK and plasmid stabilization by *ccd*, we quantified the retention of a partition-defective mini-F plasmid in lambda lysogens by flow cytometry over 40 generations. Plasmid retention was also investigated in other lambdoid lysogens (φ80 and H-19B) as well as in heteroimmune lysogens (λ_imm434_ and λ_imm21_). All lysogens showed a similar retention of the empty plasmid as the wild-type strain (Figure 4D). On the other hand, retention of a *ccd*-encoding plasmid was improved with variable efficiencies in lysogens (Figure 4D). The λ_imm21_ lysogen showed the greatest retention with 86% fluorescent cells after 4 days of culture, while an H-19B lysogen had the poorest retention with 72% fluorescent cells, compared to a non-lysogen that displayed only 66% fluorescent cells (Figure 4D). Lysogeny by lambdoid prophages therefore increased PSK by *ccd*, through the induction of a lytic cycle in plasmid-free segregants that would have otherwise repaired CcdB-induced DNA damage. Since lysogeny is common in natural *E. coli* isolates, this might provide an explanation for the relative inefficiency of the *ccd* system to kill plasmid-free segregants in the λ-deficient MG1655 lab strain. Our results therefore highlight cooperative and mutually beneficial behaviors between TA systems and prophages.

### PSK is a conserved mechanism for TA-mediated plasmid stabilization

**Figure 5.**
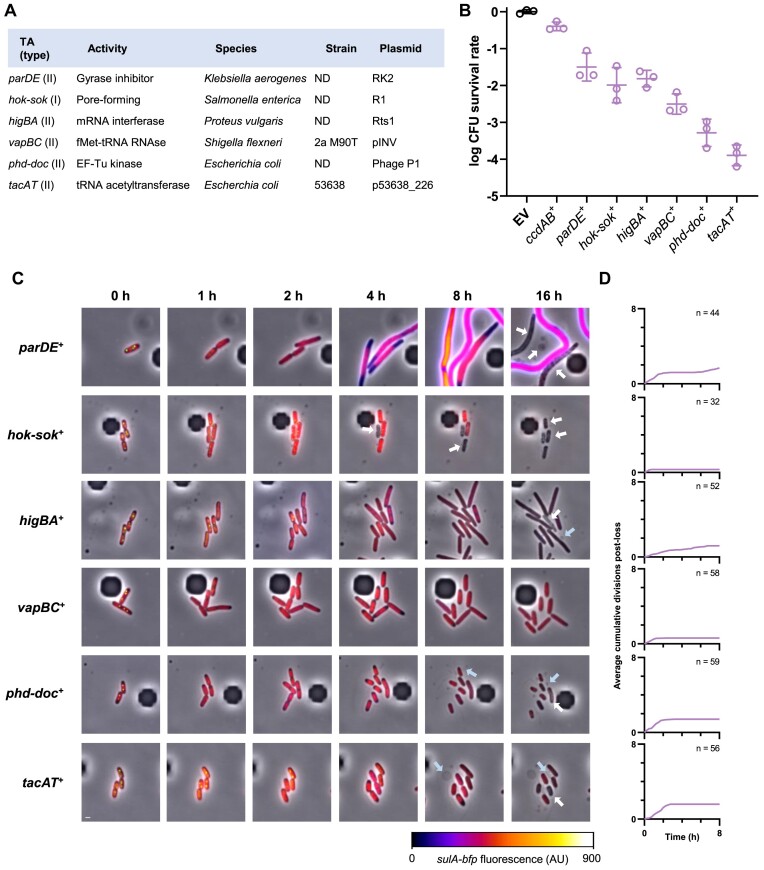
Conservation of PSK across TA families. (**A**) Recapitulative table of tested TA systems and their origin. ND: not determined. (**B**) TA-induced viability loss under plasmid curing conditions. Cells transformed with pSce and pNF04 derivatives containing the indicated TA systems were grown to exponential phase in MOPS medium containing 0.4% glucose, 25 μg/ml kanamycin and 20 μg/ml chloramphenicol, serially diluted and spotted on M9 plates containing 20 μg/ml chloramphenicol and either 0.4% glucose and 25 μg/ml kanamycin to promote plasmid retention or 0.1% glucose and 0.3% arabinose to promote plasmid curing. Data represent the geometric mean and standard deviation of three independent experiments. (**C**, **D**) Time-lapse fluorescence microscopy analysis of I-SceI-mediated plasmid curing. FN053 cells were transformed with pSce and either pNF04 (EV, top) or pNF04*ccd* (bottom) plasmids, grown on MOPS medium containing 0.4% glucose, 25 μg/ml kanamycin and 20 μg/ml chloramphenicol, and then loaded on microfluidic chips perfused with MOPS medium containing 0.4% arabinose and 20 μg/ml chloramphenicol at time 0 to induce plasmid curing. Cells were imaged by time-lapse fluorescence microscopy every 15 min, with time 0 as the last time point where foci were detected. (**C**) Representative micrographs of plasmid-free segregants following I-SceI-mediated curing. SopB-mNeongreen is shown in green, the HU-mCherry fusion that localizes the chromosome is shown in red and the *sulA-bfp* reporter is shown with the associated color scale. Scale bar is 1 μm. White arrows show loss of cytosolic content, while blue arrows show blebbing. (**D**) Quantification of divisions by microscopy after plasmid loss. The number of cumulative divisions for observed plasmid loss events was quantified at each 15 min time point after SopB foci loss and averaged over the indicated number of imaged plasmid loss events (*n*).

Although *ccd* and *hok-sok* were the first identified TA systems, other plasmid-encoded TAs with various toxic activities were subsequently identified. These include the *parDE* system from IncP-1 plasmids RK2 and RP4, in which the toxin is a DNA gyrase inhibitor structurally unrelated to CcdB (41,42), the *higBA* system from the Rts1 plasmid, in which the toxin cleaves messenger RNAs in a translation-dependent manner by entering the ribosomal A site (43,44), the *vapBC* system from the plasmid pINV of *S. flexneri*, in which the toxin cleaves the anticodon loop of the initiator Met-tRNA^fMet^ (45–47), and the *phd-doc* system from phage P1, in which the toxin inactivates the translation elongation factor Tu by phosphorylation (16,48) (Figure 5A). We also cloned a plasmid-encoded *tacAT* system that showed 90% identity at the protein level with the well-described *tacAT* system from *Salmonella enterica*, which acetylates Gly-tRNA^Gly^ (49) (Figure 5A). So far, killing by these systems has only been studied using toxin overproduction, with the assumption that activation of toxins from their native loci during PSK would trigger comparable toxicity and killing (3,41,43,45,48). As with *ccd*, these systems inhibited growth at non-permissive temperatures when cloned in temperature-sensitive plasmids, suggesting that these systems are activated when plasmid replication is compromised (16,42,44,47).

We therefore assessed whether these systems could trigger PSK when produced through their native loci by cloning them in our curable mini-F vector. Curing plasmids encoding each of these six TAs reduced viability by several orders of magnitude, suggesting that these TAs kill plasmid-free segregants more efficiently compared to *ccd* under our experimental conditions (Figure 5B). Interestingly, these systems showed varying efficiencies of killing, with *vapBC* (3.6 × 10^−3^), *phd-doc* (6.4 × 10^−4^) and *tacAT* (1.4 × 10^−4^) displaying the lowest survival rates (Figure 5B). On the contrary, *parDE* (4.2 × 10^−2^), *hok-sok* (1.5 × 10^−2^) and *higBA* (1.7 × 10^−2^) showed lower killing efficiencies after curing, suggesting that these systems are less efficient at killing plasmid-free segregants (Figure 5B).

Microscopy analysis of plasmid-cured cells revealed that plasmid loss is correlated with division inhibition for all tested TA systems (Figure 5C and D, and Supplementary Movies S5–S10). Hours-long delays occur between plasmid curing and division inhibition for type II systems (Figures 3B and 5D), which is consistent with reported half-lives in the hour range reported for CcdA, ParD and Phd proteic antitoxins (17,18,50). On the other hand, growth inhibition by Hok occurred only 30 min after foci loss (Figure 5D), consistent with the short half-life of 3–4 min previously described for the *sok* RNA antitoxin (51).

After plasmid curing, tested TA systems induce phenotypes consistent with their associated activities. The *parDE* system, which poisons DNA gyrase, induced similar phenotypes as the *ccd* system, including induction of the SOS response and filamentation (Figure 5C and Supplementary Movie S5). The *hok-sok* system induced rapid growth arrest followed by congregation of SopB-mNeongreen fluorescence on the edges of the cells. Loss of cytoplasmic content could be detected over the course of several hours, consistent with the previous observation of Hok-induced ‘ghost’ cells (7) (Figure 5C, Supplementary Figure S5 and Supplementary Movie S6). Translation-inhibiting TAs (*vapBC*, *phd-doc*, *higBA* and *tacAT*) induced growth arrest, although *higBA* plasmid-free segregants kept dividing at a slow pace throughout the experiment (Figure 5C and Supplementary Movies S7–S10). Surprisingly, blebbing-like protrusions visible by phase contrast could be detected in *phd-doc*, *higBA* and *tacAT* plasmid-free segregants, suggesting that the toxins of this systems could perturb envelope homeostasis by an unknown mechanism (Figure 5C, Supplementary Figure S5, and Supplementary Movies S7, S9 and S10). Altogether, these results show that activation of various families of type I and II TA systems from their native loci results in cell death, demonstrating that PSK is a conserved mechanism by which TA systems mediate plasmid addiction.

## Discussion

In this work, we designed a system that enables curing of plasmids as a mean to study PSK by TA systems. Visual clues of PSK were obtained in previous studies by imaging cultures several hours after destabilizing TA-encoding replicons, with *ccd* inducing filamentation and production of anucleate cells, and *hok-sok* inducing loss of cytosolic content (7,11). Likewise, destabilization of chromosome II (ChrII) in *Vibrio cholerae* through the deletion of its *parABS* system resulted in toxicity and DNA damage, which were dependent on three ChrII-encoded *parDE* systems (52). However, live imaging of PSK has never been reported. Here, we report the first live observation of this phenomenon on its full scale and in a systematic manner.

Our work first imaged thousands of cell divisions and plasmid partition events by microscopy, showing that the *ccd* system does not increase segregational stability of its mini-F replicon. Rather, loss of a plasmid that carried the *ccd* system induces the SOS response and cell filamentation, supporting earlier observations that *ccd* leads to DNA damage in plasmid-free segregants. To facilitate the study of such TA-induced phenotypes, we were able to induce quasi-synchronous curing of the mini-F plasmid in the whole population through I-SceI-mediated cleavage of the plasmid, allowing to visualize intoxication of plasmid-free segregants by CcdB. A significant portion of plasmid-free segregants were able to survive PSK by repairing CcdB-mediated DSBs, which translated into a poor stabilizing capacity for this system. Accordingly, previous reports showed that *ccd* is a poor plasmid stabilizer compared to other TA systems like *vapBC*, *parDE* or *hok-sok* that promoted plasmid retention orders of magnitude higher than *ccd* (46,53). Redundancy of *ccd* with other TA systems encoded on the same plasmid, *i.e*.*flm* (which is quasi-identical to *hok*-*sok*) and *srn* on F, as well as *vapBC* and *gmvAT* on pINV (46,54), could lead to a partial decay in the activity of the *ccd* system, through mutations that reduce *ccd* expression or diminish CcdB binding to DNA gyrase. Plasmid hosts could also have acquired mutations in the *gyrA* gene that reduce CcdB toxicity to facilitate plasmid curing and/or reduce lethality induced by *ccd*. There has recently been increasingly strong evidence that TA systems provide defense against phages through abortive infection (*abi*) (6). While our observations do not exclude that canonical type II systems like the ones we studied here would participate in this phenomenon, we believe that these systems do not induce *abi*. A first argument discussed by LeRoux and Laub states that most type II antitoxin half-lives are too long to enable antitoxin depletion during the life cycle of a phage (20–40 min) (6). Indeed, our observations support this idea since growth inhibition by type II toxins can only be observed at least 1 h after activation of these systems by plasmid curing (Figures 3B and 5D). Moreover, our data show that activation of the *ccd* system induces the lytic cycle of lambdoid prophages without hindering phage production or inducing *abi* (Figure 4). This induction of the lytic cycle increased the lethality of *ccd* activation in plasmid-free segregants, hinting at possible cooperative behaviors between prophages and plasmid-encoded TA systems (Figure 4).

By using I-SceI-mediated curing of mini-F vectors encoding several other TA systems, our work demonstrates that PSK is a conserved mechanism by which TAs of type I and II systems stabilize their replicon. Time-lapse microscopy analysis allowed us to track single plasmid curing events, which lead to defects consistent with the activities of each tested system, whether it be topoisomerase poisoning, pore forming or translation inhibiting. While inhibiting translation using antibiotics (*e.g*. tetracyclines, aminoglycosides, macrolides and phenicols) is widely regarded as bacteriostatic (55,56), in our experimental setup, translation-inhibiting TAs induced killing as or even more efficiently compared to TAs whose activities can be regarded as bactericidal, such as DNA gyrase poisoning or inner membrane permeation. Interestingly, translation-inhibiting TAs induce cell lysis (*higBA*) or envelope abnormalities (*phd-doc*, *tacAT*). While the activity of these translation-inhibiting toxins has been regarded as reversible (55,57), our results suggest that activation of plasmid-encoded TA systems leads to irreversible cell death, with the corruption of translation machinery by these systems having secondary effects that affect envelope homeostasis, which are ultimately bactericidal. However, the molecular mechanisms underlying these secondary effects remain to be elucidated. While non-lethal mechanisms have been proposed to explain TA-mediated plasmid stabilization (21,22), PSK is not mutually exclusive with these. It is therefore plausible that some TA systems stabilize plasmids by cumulating PSK with other control mechanisms to maximize their retention.

While the effects of toxin on bacterial physiology are studied using multicopy expression vectors (*e.g*. pBAD arabinose-inducible plasmids), which enable the ectopic production of biologically irrelevant levels of toxin (3), the experimental setup we describe in this study allows to study intoxication by TA systems and its downstream effects by triggering these systems through their canonical activation pathway, *i.e*. through the loss of TA-encoding genes. We present here that our setup can be used to study TA activation at the single-cell level to assess morphological parameters as well as the activation of responses using fluorescent reporters. Unlike previously used setups used to study PSK that rely on plasmid replication inhibition and were therefore dependent on cell division, synchronous plasmid curing in the whole population as presented here enables the study of toxin effects at the population level using bulk experiments. For example, global approaches like RNA sequencing, ribosome profiling or RNA end mapping would be well suited to study the effect of translation-inhibiting toxins at biologically relevant toxin levels. Other biological parameters such as accumulation of reactive oxygen species, ATP levels, and membrane integrity and polarization can be assessed to study the downstream effects of these toxins that ultimately result in cell death.

## Supplementary Material

gkae018_Supplemental_Files

## Data Availability

The data underlying this article are available in the article and in its online supplementary material.

## Notes

Present address: Nathan Fraikin, Molecular Microbiology and Structural Biology (MMSB), Université Lyon 1, CNRS, UMR 5086, Lyon, France.
