# Supplementary figures and images for: Single-cell evidence for plasmid addiction mediated by toxin–antitoxin systems
Source: Nucleic Acids Res. 2024 Jan 15;52(4):1847–59. doi: 10.1093/nar/gkae018 (PMC10899753; doi:10.1093/nar/gkae018)

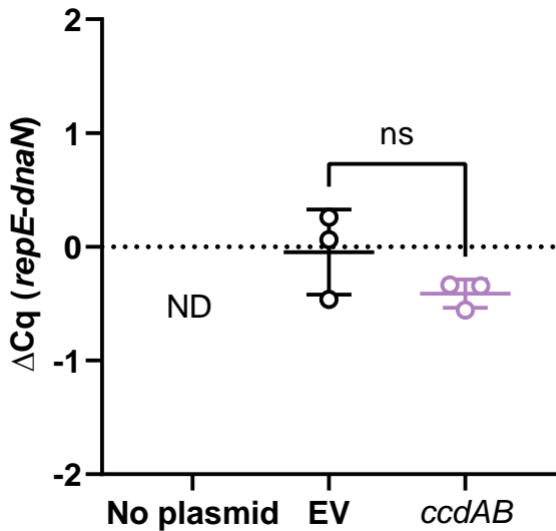

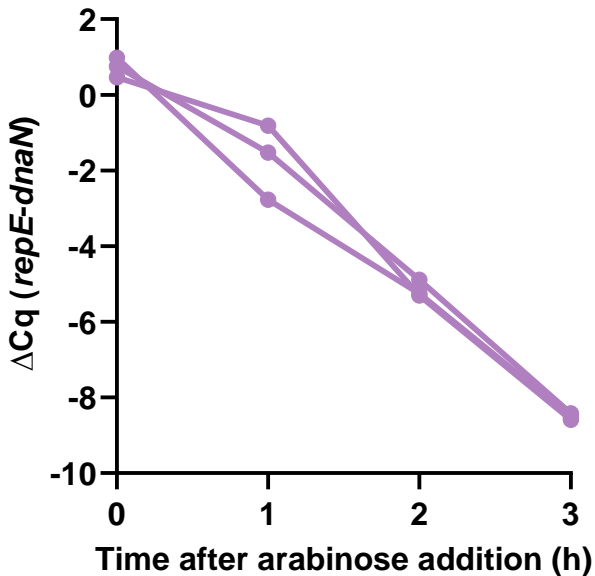

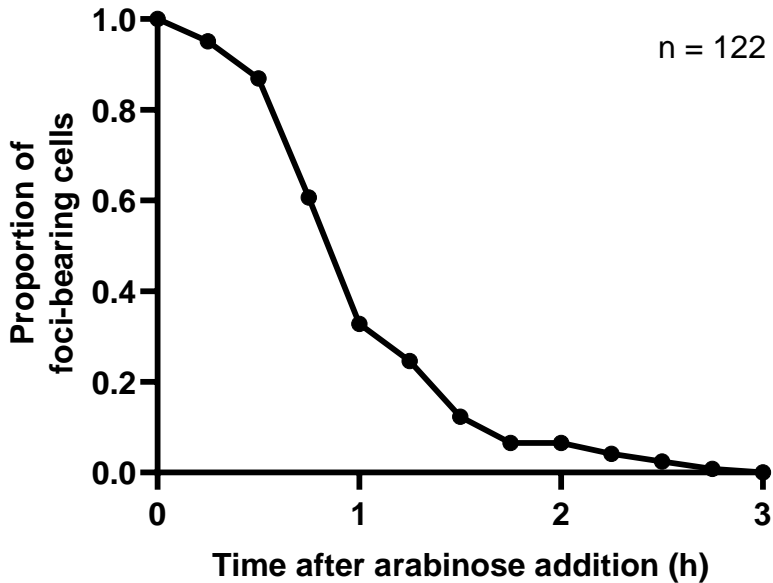

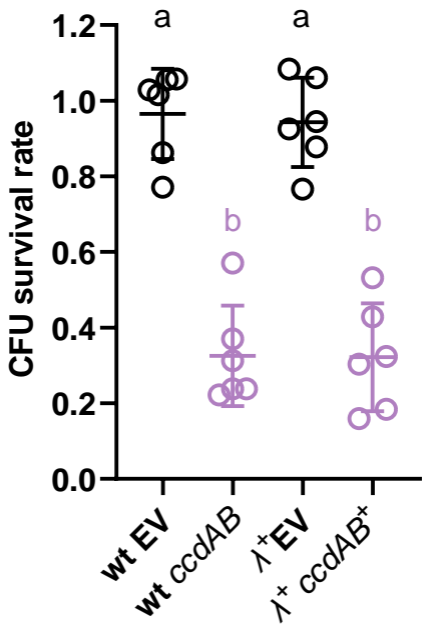

*phd-doc*<sup>+</sup>

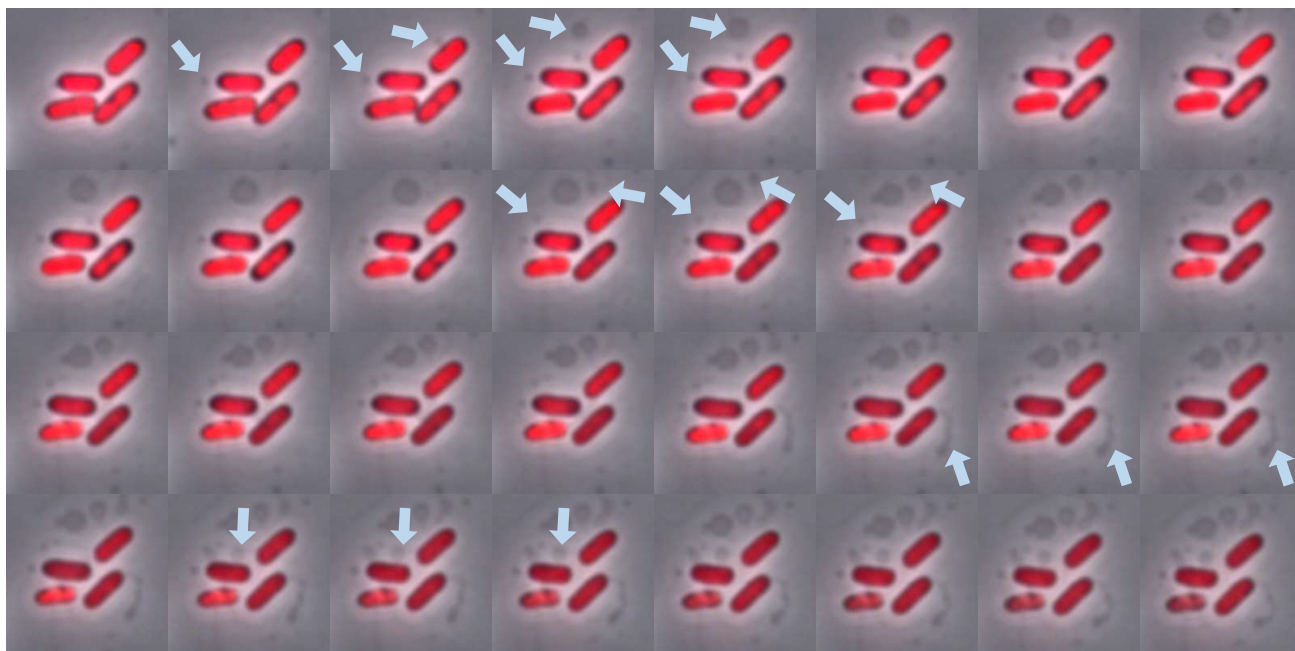

*hok-sok*<sup>+</sup>

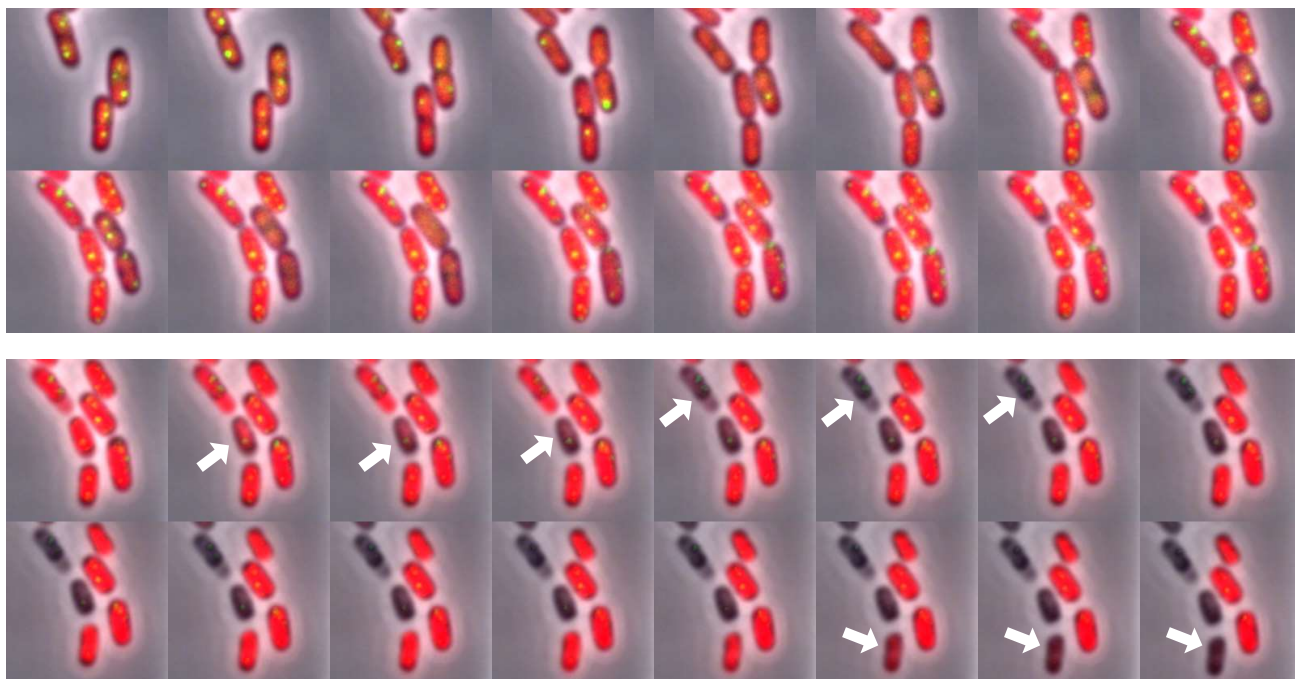

*tacAT*<sup>+</sup>

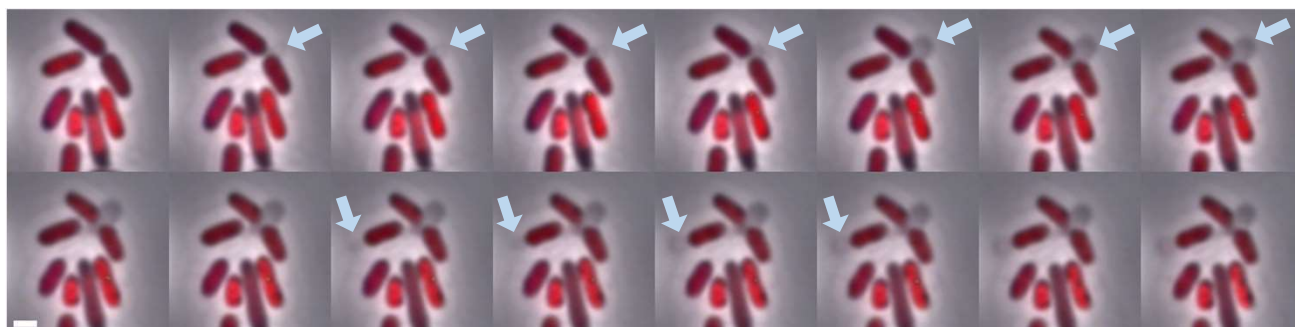

Supplement: gkae018_Supplemental_Files [file gkae018_supplemental_files.zip › Supplementary figures.pdf]
